# Reframing Nutritional Microbiota Studies To Reflect an Inherent Metabolic Flexibility of the Human Gut: a Narrative Review Focusing on High-Fat Diets

**DOI:** 10.1128/mBio.00579-21

**Published:** 2021-04-13

**Authors:** Jonathan Sholl, Lucy J. Mailing, Thomas R. Wood

**Affiliations:** a Université Bordeaux, CNRS, ImmunoConcEpT, UMR 5164, Bordeaux, France; b Independent Researcher, Milwaukee, Wisconsin, USA; c Institute for Human and Machine Cognition, Pensacola, Florida, USA; d Center on Human Development and Disability, University of Washington, Seattle, Washington, USA; e Department of Pediatrics, University of Washington, Seattle, Washington, USA; University of Texas Health Science Center at Houston

**Keywords:** cancer, gut health, high-fat diets, metabolic flexibility, microbiota

## Abstract

There is a broad consensus in nutritional-microbiota research that high-fat (HF) diets are harmful to human health, at least in part through their modulation of the gut microbiota. However, various studies also support the inherent flexibility of the human gut and our microbiota’s ability to adapt to a variety of food sources, suggesting a more nuanced picture. In this article, we first discuss some problems facing basic translational research and provide a different framework for thinking about diet and gut health in terms of metabolic flexibility. We then offer evidence that well-formulated HF diets, such as ketogenic diets, may provide healthful alternative fuel sources for the human gut. We place this in the context of cancer research, where this concern over HF diets is also expressed, and consider various potential objections concerning the effects of lipopolysaccharides, trimethylamine-*N*-oxide, and secondary bile acids on human gut health. We end by providing some general suggestions for how to improve research and clinical practice with respect to the gut microbiota when considering the framework of metabolic flexibility.

## Abstract (precis)

There is a broad consensus in nutritional-microbiota research that high-fat (HF) diets are harmful to human health, at least in part through their modulation of the gut microbiota. However, various studies also support the inherent flexibility of the human gut and our microbiota’s ability to adapt to a variety of food sources, suggesting a more nuanced picture.

## INTRODUCTION

It is generally accepted that diet is a major factor shaping both the composition and the function of the human gut microbiota. However, much debate focuses on the health effects of dietary components, with fiber generally being seen as not only beneficial but necessary and animal fat (and sometimes protein) from “high-fat (HF) diets” being singled out as detrimental to the gut microbiota (1–9). As a result, concerns over HF diets feature heavily in the framing of studies on the microbiota and health. For instance, HF or even “high-protein, low-carbohydrate” diets are often suggested to play a causal role in various forms of cancer, cardiovascular disease, immunological dysregulation, and diabetes, through a variety of mechanisms (10–14). This concern is expressed by international authorities on gut health, e.g., the European Society of Neurogastroenterology and Motility (15), and in consensus statements by groups like the International Cancer Microbiome Consortium (16).

It seems safe to say that the consensus is that HF diets are harmful to human health, at least in part through their modulation of our gut microbiota. Put differently, the primary substance that feeds “beneficial” gut microbes is “microbiota-accessible carbohydrates” (17), and in the absence of these, protein and fat will deteriorate our gut health. One of the most cited studies used to support this consensus is that of David et al. (18). While this study demonstrates how quickly the human gut microbiota adapts to dietary changes, what is less clear is how this should be interpreted (9). As we will discuss, this study highlights the need to consider the metabolic flexibility of the gut (19, 20). We are still far from being able to precisely define a “healthy” gut microbiota (21–25), and it is quite likely that the human gut and its microbial symbionts evolved to adapt to a variety of macronutrient patterns. Acknowledging this flexibility will help to expand research and guide clinical interventions.

Here, we suggest one way in which translational research on nutrition and the microbiota can be misleading (1st section) and provide a way to reframe this research in terms of metabolic flexibility (2nd section). We then offer evidence supporting the potential healthfulness of alternative fuel sources derived from HF ketogenic diets (KDs) (3rd section) and question the harmful role of these diets in diseases such as cancer (4th section). After addressing some likely objections (5th section), we end by raising the concern that the consensus on dietary fat may reflect a research bias more than physiological reality and provide suggestions for future research.

## LOST IN TRANSLATION: OF MICE AND JUNK FOOD

We are not the first to point out the limitations of preclinical nutritional microbiota research or the ubiquitous problem of HF diets in animal models. These diets are typically composed of soybean oil, lard, refined sugar, and little to no fiber (26, 27), which Craig Warden called “the mouse equivalent of pork rinds, ribs, and coke” (28). The classic animal HF diet is therefore much more reflective of the standard American diet than nutritionally replete high-fat diets, such as therapeutic KDs (29, 30). Evidence for the role of simple sugars in harmfully disrupting the gut microbiota is growing (31), and this alone should provide ample reason not to draw conclusions based solely on fat content without considering other macronutrients or dietary quality.

While human metabolism can adapt to diets higher in either fats or carbohydrates, the natural diet of a mouse is low in fat and high in carbohydrates. It is therefore unsurprising that mice develop issues when eating a species-inappropriate diet. The strain of mice commonly used for such studies, C57BL/6, has also been genetically selected for its ability to gain weight in response to a HF diet. While humans are capable of weight loss or gain on a variety of dietary patterns (32–34), C57BL/6 mice have greater weight gain and metabolic disruptions on low-carbohydrate diets (35). Consequently, “…rodent models of obesity may be most valuable in the understanding of how metabolic mechanisms can work in ways different from the effect in humans” (35). Broadly translating findings from inbred mice fed a highly refined HF diet to humans is therefore fraught with potential for misunderstanding.

Similar problems exist in the clinical literature examining effects of the diet on the gut microbiota and associated disease risk. For instance, in the Malmö Offspring Study, Ericson et al. identified “health-conscious” and “sugar and high-fat dairy” dietary patterns associated with decreased and increased risk of having prediabetes, respectively (36). The latter pattern was characterized by high intakes of pastry/desserts, high-fat milk/cream, low-fiber bread, potatoes, and processed/red meat, with the overarching assumption that these components all equally and significantly contribute to the potential negative effects of this dietary pattern. Though these foods may cluster together frequently on the population level, we cannot assume that they contribute equally to any associated health outcomes since each of them would be expected to have very different effects on both the gut microbiota and on general health. As highlighted in the commentary accompanying reference 36, the association between the health-conscious dietary pattern and prediabetes was lost after adjusting for body mass index (BMI) (2), suggesting that a primary driver of differences between dietary patterns may be caloric intake. Any attempt to assess the effects of dietary components on health must consider food processing and energy density, both of which appear to contribute to increased caloric intake beyond the effect of individual macronutrients (37, 38). One must also consider whether it is the presence or absence of certain foods that drives downstream effects (2). Without a nuanced approach examining dietary quality and individual dietary components, we are left making assumptions about fat in human diets similar to those made when we attribute the effects of HF diets in rodents purely to fat content.

We largely agree that the “Western” diet full of processed food causes problems for both the mouse and the human gut microbiota. However, there are a variety of ways to construct an HF diet, with data from humans suggesting that well-formulated therapeutic KDs, which in some clinical trials contain between 3 and 5 servings of nonstarchy vegetables per day (39, 40), may be more beneficial for our gut and overall health than some animal studies suggest (30, 41, 42). What is needed, then, is a way to reframe the debate to better reflect the overall evidence.

## REFRAMING A HEALTHY GUT IN TERMS OF EVOLVED FLEXIBILITY

New technologies and greater interest in gut health in recent years have dramatically increased our understanding of gut microbes. Nevertheless, we are still unable to define a “healthy” gut microbiota (22, 25). On average, any two individuals share only about a third of their gut microbiota, with the other two-thirds varying depending on genetics, geographical location, history of antibiotic and medication use, mode of delivery at birth, diet, and other undetermined factors (43, 44). It is even possible that two otherwise-healthy individuals can show no overlap in microbiota composition (44). Thus, outside clear instances of dysbiosis, we have insufficient information to say that one individual’s “two-thirds” is any better than another’s.

While it is generally believed that diversity and community stability are key components of a healthy gut ecosystem, even these can sometimes be associated with diseased states (45, 46). Some of the keystone microbes commonly considered crucial for gut health, such as *Bifidobacterium*, are completely absent from the guts of traditional cultures, like the Hadza, who are otherwise virtually free of chronic disease (47). Gut health and dysbiosis thus remain vague and sometimes contested concepts (25, 48, 49); if there is a healthy “core” microbiota, it may be at the level of microbial functions, not species (23, 43). Part of this relative lack of insight may result from technologies such as 16S rRNA sequencing, which do not provide accurate information beyond the genus level and provide little insight into microbial functions (50). While there is hope that higher-resolution technologies (e.g., metagenomics, metabolomics), larger data sets, and advanced computing techniques will bring us closer to defining a healthy microbiota, many researchers call for moving away from cataloguing species and toward an approach that considers the intricate nature of microbiota-host interactions (22, 51).

While technological advances are eagerly awaited, some initial clarity might come from placing the human microbiota in its evolutionary context. Our relationship with our gut microbes is the product of thousands of generations of close coevolution (52, 53). The environments in which we evolved also required regular adaptation to changing conditions. Our ancestors may not always have had steady access to food and would likely have undergone occasional bouts of significant deprivation when food was scarce (54, 55). Similarly, diets changed seasonally and geographically, as is reflected by the seasonal changes in the guts of traditional populations, like the Hadza (56), or in the specific adaptations in cultures known to eat relatively few plant foods, such as the Inuit (57, 58). This variability can be explained in terms of metabolic flexibility (19), which is the evolved ability to shift our metabolism to changes in dietary intake: to burn and use carbohydrates when they are plentiful and to turn dietary fat or stored body fat into ketones for energy when food or carbohydrates are scarce. Consequently, it seems likely that our guts also exhibit the flexibility to adapt to changing food sources rather than suffer significant gut dysfunction whenever fiber is absent.

In line with this evolutionary perspective on the compositional and functional adaptability of our gut and its microbiota, David et al. write (18),

Our findings that the human gut microbiome can rapidly switch between herbivorous and carnivorous functional profiles may reflect past selective pressures during human evolution. Consumption of animal foods by our ancestors was likely volatile, depending on season and stochastic foraging success, with readily available plant foods offering a fallback source of calories and nutrients. Microbial communities that could quickly, and appropriately, shift their functional repertoire in response to diet change would have subsequently enhanced human dietary flexibility.

In other words, a “healthy” gut microbiota adapts to a wide range of food sources and does not necessarily become more or less pathogenic depending on the amount of carbohydrate or fat in the diet. Moreover, while short-term dietary changes tend to produce significant changes in the gut microbiota (18), long-term studies suggest a relative resilience of the microbiota to shifts in diet (59). Due to the aforementioned factors shaping gut microbiota (43, 44), we should consider whether a dietary change must produce significant physiological changes in the host before a new microbial stability is achieved, with diet-induced fluctuations merely an expression of the gut’s ability to adapt to ensure optimal function. If human guts are inherently metabolically flexible, short-term diet-induced changes in microbiota composition could be considered a potential hallmark of gut health (60). We should thus determine whether the short- and long-term taxonomic changes resulting from this metabolic flexibility are predictive of overall health outcomes and how/whether the microbiota drives those outcomes (61–63).

## EVIDENCE FOR HIGH-FAT KETOGENIC DIETS—CONSIDERING ALTERNATIVE PATHWAYS

Gut bacteria metabolize complex carbohydrates to produce short-chain fatty acids (SCFAs), like acetate, propionate, and butyrate, with the last being the preferred fuel source for gut epithelial cells. Published estimates suggest that butyrate provides about 70% of colonic epithelial cell energy requirements (64), with a regular supply of butyrate required to maintain gut barrier function. What remains to be seen is how different diets modulate SCFA production and whether this results in different downstream health effects.

### Animal-based diets.

The work of David et al. (18) has been instrumental in highlighting how quickly and reliably the human gut microbiota adapts to dietary changes. What is unclear is whether this study should be used to support the avoidance of diets high in fat or protein. Ten healthy human volunteers were placed on a short-term plant-based diet (PBD) consisting of 300 g of carbohydrate per day from cereal, vegetables, rice, lentils, and fruit or on an animal-based diet (ABD) consisting of less than 3 g of carbohydrates per day, with 30% of calories from protein and 70% of calories from fat from eggs, meat, and cheese. Subjects on the ABD group were confirmed to be in ketosis by day 2 of the diet, with distinct gut microbial communities emerging in both diet groups within 3 days.

**FIG 1:**
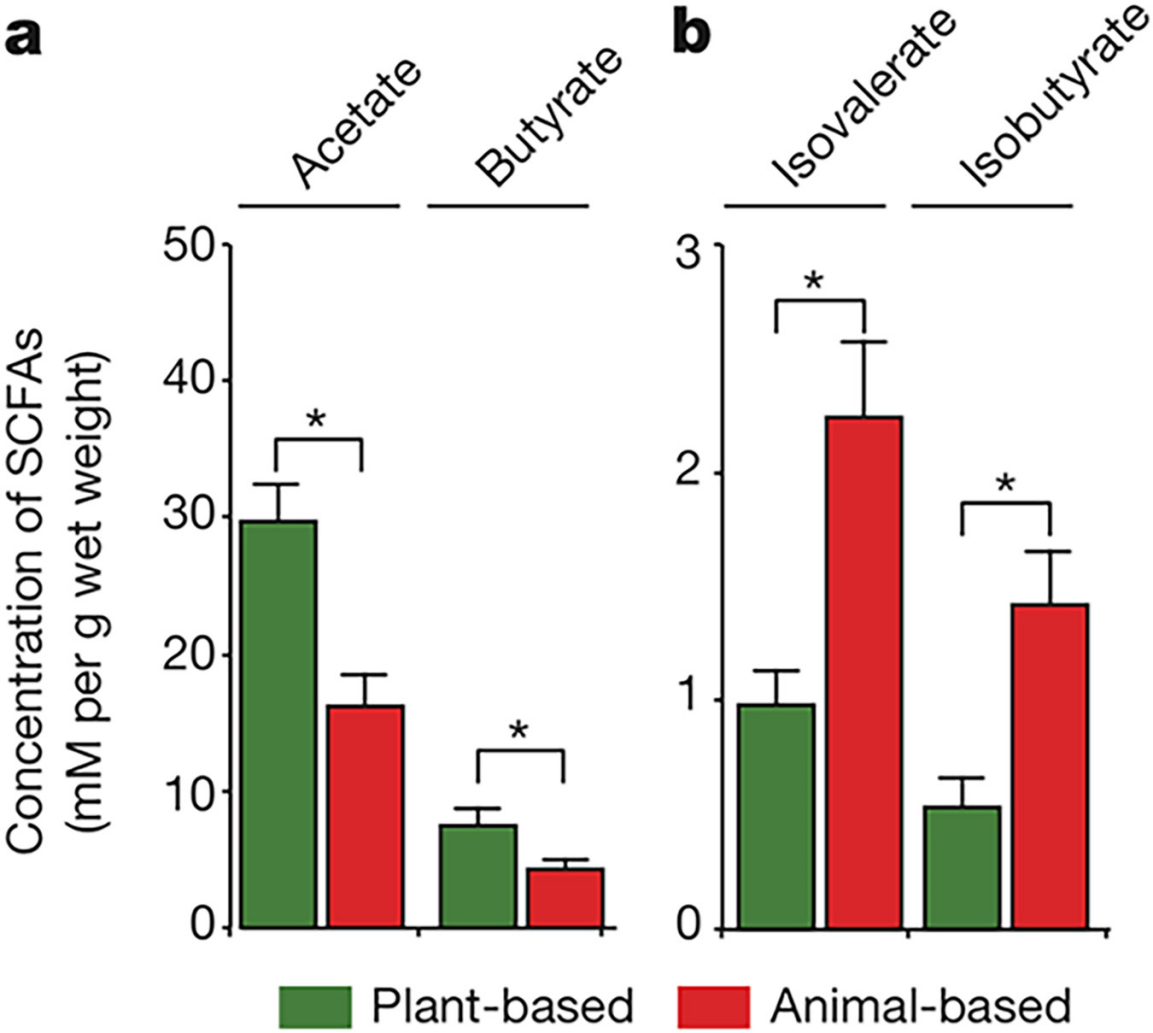
Short-chain fatty acid (SCFA) production in humans eating low-fat, plant-based and high-fat, animal-based diets. (a) Plant-based diets result in roughly twice the production of acetate and butyrate. (b) Animal-based diets result in roughly twice the production of isovalerate and isobutyrate, which have metabolic functions overlapping those of the traditional SCFAs acetate and butyrate. (Republished from reference 18 with the permission of the publisher.)

The most interesting and perhaps contentious finding was that there was no significant change in alpha-diversity in either group (18). Those on the ABD saw an increase in the relative abundance of bile-tolerant microorganisms, like *Bilophila*, *Alistipes*, and *Bacteroides* spp., and a decrease in the relative abundance of microbes known to metabolize complex dietary plant fibers, such as *Roseburia*, *Eubacterium*, and *Ruminococcus* spp. While often cited as evidence that an ABD is harmful, this is far from conclusive. The PBD, despite being supposedly uniquely capable of producing butyrate from microbiota-accessible carbohydrate metabolism, produced only slightly more butyrate than did the ABD, with the ABD also resulting in significantly greater production of isovalerate and isobutyrate (Fig. 1) (18). Isobutyrate has been shown to activate many of the same receptors as butyrate (see “Considering alternative pathways” below), weakening the notion that PBDs are significantly “better” for the gut due to butyrate/SCFA production.

### Mucus barrier.

Another recent study by Ang et al. confirmed that a ketogenic diet (KD) can alter the structure and function of the gut microbiota (65). In humans, among the most significant changes of the fecal microbiota following a KD was a dramatic reduction in the abundance of several *Bifidobacterium* species. In controlled-feeding studies of mice, the researchers found that KDs had a unique impact on the gut microbiota relative to conventional HF diets, with the abundance of *Bifidobacterium* organisms decreasing with increasing carbohydrate restriction. Further experiments found that both a KD or ketone ester supplementation increased beta-hydroxybutyrate (βHB) in the lumen of the gut and in colon tissue (65), with ketone bodies directly inhibiting the growth of *Bifidobacterium*. Ketosis was also associated with a reduction in small intestinal Th17 cells, which help maintain the gut mucosal barrier and contribute to pathogen clearance at mucosal surfaces. However, Th17 cells have also been implicated in autoimmune and inflammatory disorders (66).

Next, Ang et al. (65) sought to determine whether the change in Th17 cells was dependent on the ketone-induced changes in the microbiota. Mice that received a fecal transplant of the ketone-fed microbiota from human donors had significantly fewer intestinal Th17 cells. Contrary to previous findings that mice fed fiber-free diets had a significant breakdown of the colonic mucus layer (67, 68), Ang et al. write, “A ketogenic diet maintains a robust mucus layer despite the lack of fermentable carbohydrates” (65). The KD maintained not only the thickness of the mucus layer but also the expression of Muc2, the primary constituent of gut mucus. Nutritional ketosis might actually support the gut mucus layer.

### Multiple sclerosis and epilepsy.

There are various levels of support for therapeutic KDs on the gut and overall health in longer-term studies, for instance, the long-term effects of a KD on the fecal microbiota in 25 patients with multiple sclerosis (MS) (69). Like many autoimmune diseases, MS is associated with gut pathologies, with gut dysbiosis and intestinal permeability potentially preceding the development of autoimmunity (70). Swidsinski et al. (69) found that patients with MS tended to have reduced numbers of *Roseburia*, *Bacteroides*, and Faecalibacterium prausnitzii organisms at baseline than healthy individuals. The effects of a 6-month therapeutic KD were biphasic: “In the short term, bacterial concentrations and diversity were further reduced. They started to recover at week 12 and exceeded significantly the baseline values after 23 to 24 weeks on the ketogenic diet” (69). Such studies are inconclusive since they are relatively uncontrolled, but they nevertheless further support the need to consider the time course of dietary adaptation before determining whether a diet is beneficial or detrimental for the gut microbiota.

In another context, researchers investigated whether the beneficial effects of a therapeutic KD on epilepsy are mediated through the gut microbiota (71). The KD reduced microbial diversity but increased the abundance of Akkermansia muciniphila and *Parabacteroides* spp. By treating mice fed a normal high-carbohydrate diet with these specific microbes, the researchers demonstrated that these taxa were at least partly responsible for the antiepileptic effects of the KD. Similar microbial changes, as well as increases in butyrate and propionate, have been observed when using a modified Mediterranean ketogenic diet in Alzheimer’s patients (72).

Together with the David et al. study (18), these findings suggest that, while short-term dietary changes can rapidly shift the composition of the gut microbiota, these changes may not be detrimental and may provide benefit. They also underline the need to look at long-term dietary changes and collect samples at multiple time points to determine the true effect of an intervention like a therapeutic KD, including whether the benefits or any risks are mediated through changes in the microbiota.

### Considering alternative pathways.

Multiple strands of evidence question an assumption about “normal” metabolic pathways in the gut. Alongside the SCFAs mentioned above, there are several other molecules that can serve as sources of fuel for gut epithelial cells. The very idea of a “preferred” fuel source may be skewed from studying people (and rodents) who eat a large amount of microbiota-accessible carbohydrates. In other words, while butyrate production may be reduced on a KD, other molecules can potentially take butyrate’s place to help maintain gut barrier function.

This shift to an alternative pathway is what we might expect from the perspective of metabolic flexibility, where we see a potential analogy between the butyrate-gut connection and glucose-brain connection. While glucose is a necessary fuel for the brain, we have known for some time that in the (relative) absence of carbohydrates, the body will shift its metabolism from glucose to fatty acids, producing ketone bodies, such as βHB, to support brain metabolism (73). This kind of fatty acid-based metabolism appears to have numerous neurological benefits and may be a “preferred” fuel for the brain during both development (74) and aging (75). Similarly, while researchers repeatedly stress that gut epithelial cells are uniquely fueled by the butyrate produced by our resident microbiota after consuming “microbiota-accessible carbohydrates” (17), here too the body is flexible, with βHB also being capable of supporting energy requirements in the gut.

**FIG 2:**
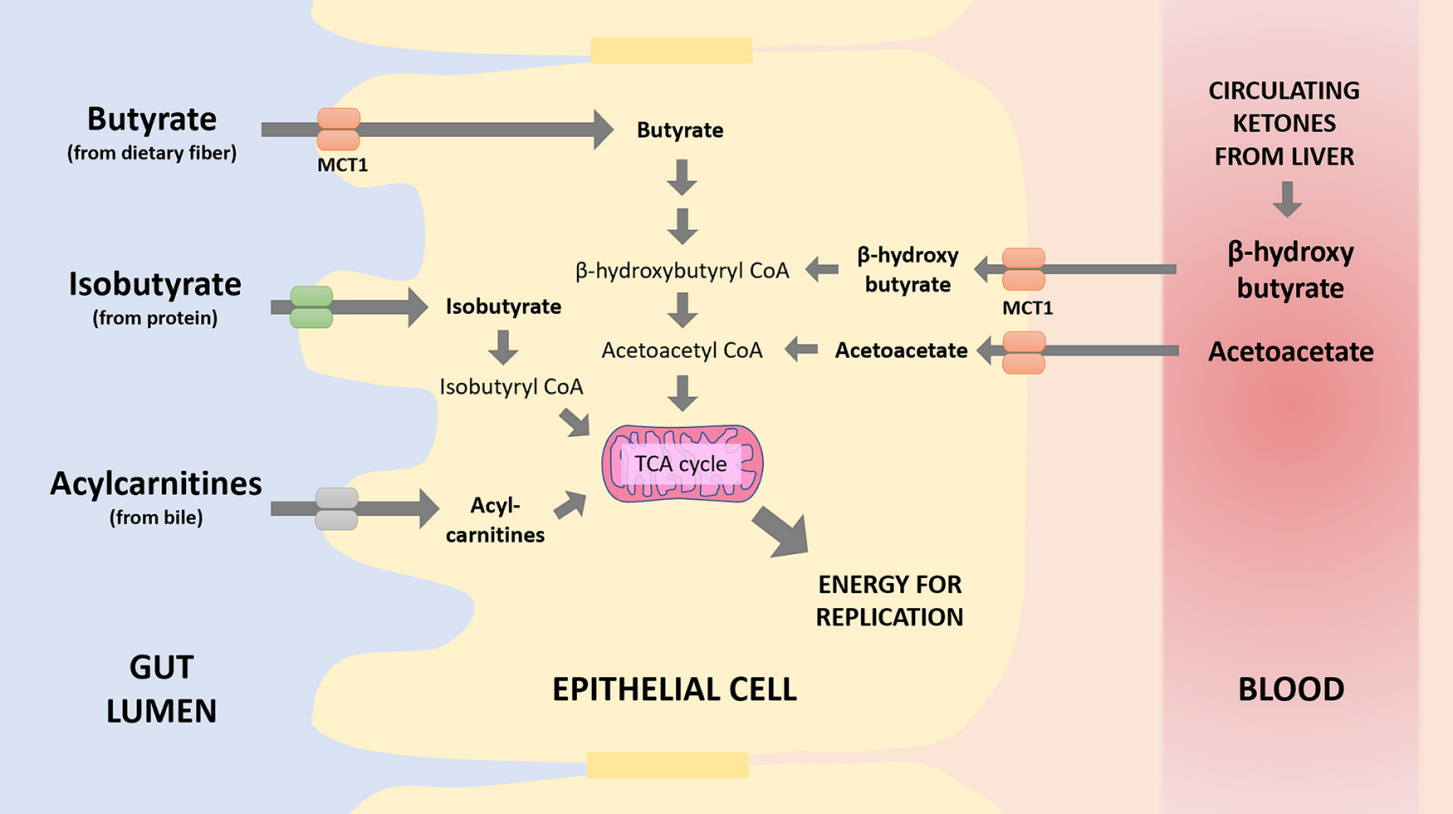
The many substrates and pathways that contribute to energy production in the intestinal epithelium. TCA, tricarboxylic acid.

In fact, there are at least four molecules that can replace butyrate: isobutyrate, acetoacetate, βHB, and bile-derived acylcarnitines (Fig. 2). Isobutyrate is a metabolite of protein fermentation that is typically produced at lower levels than butyrate. When butyrate is less abundant, isobutyrate can be absorbed from the gut lumen by gut epithelial cells and metabolized for energy (76). Fecal isobutyrate was found to be elevated in humans consuming a KD (18). Moreover, isobutyrate can stimulate the same receptors as butyrate in the gut (GPR41, GPR43, and GPR109a) to influence mucus secretion, antimicrobial peptide release, and immune regulation (77), and while isobutyrate may be produced at lower levels on a moderately high-protein diet than butyrate would be produced on a high-carbohydrate diet (18), isobutyrate appears to be a more potent stimulator of butyrate receptor GPR41 (FFAR3) than butyrate itself (78). In other words, what isobutyrate lacks in concentration relative to butyrate, it may make up for in potency. Relatedly, this may provide a reason not to confuse high-protein and refined HF diets, since the most abundant end products of protein fermentation or catabolism are SCFAs, such as isobutyrate (79). As suggested by David et al., it might be the overall context in which protein fermentation occurs that is important to downstream health outcomes rather than the protein fermentation itself.

Like butyrate, βHB can also stimulate GPR109a, reducing intestinal inflammation (80, 81). Most notably, however, both βHB and its related ketone body acetoacetate are intermediates in the pathway for butyrate metabolism; when butyrate is taken up by gut epithelial cells, it is converted into βHB first and then acetoacetate before being broken down further for energy (Fig. 2) (82). Gut epithelial cells express the monocarboxylate transporter MCT1, a primary ketone body transporter on the basolateral surface (83), and several papers suggest that gut epithelial cells are capable of utilizing ketone bodies from the vascular bed (84, 85). As gastrointestinal inflammation and mucosal damage can impair butyrate uptake from the intestinal lumen (86, 87), circulating ketones may provide a potential therapeutic option in certain patients with gastrointestinal disease.

To our knowledge, no studies have assessed the effects of ketones or a KD on gut barrier function. However, a recent study found that ketone body signaling regulates the normal function of intestinal stem cells (ISC) and their ability to respond to injury (88). In this study, β-hydroxy β-methylbutyryl-coenzyme A (HMG-CoA) synthase 2 (HMGCS2), a rate-limiting step in ketone production, was enriched in small intestinal stem cells. Ablating the *Hmgcs2* gene in mice diminished βHB levels in the crypts, compromising ISC function and regeneration of the gut epithelium after injury. Exogenous βHB rescued ISC function and partially restored intestinal regeneration. A KD also increased HMGCS2 expression and ISC number, function, and postinjury regeneration. In contrast, a glucose-supplemented diet suppressed ISC ketogenesis and skewed the differentiation of ISC toward goblet and Paneth cells. Notably, once stem cells had differentiated into mature epithelial cells and migrated out of the crypt, they expressed very little HMGCS2. This suggests that mature epithelial cells do not possess the ability to generate large amounts of ketones through the classical ketogenic pathway (via condensation of two molecules of acetyl-CoA), though we know that they have the ability to utilize ketones.

Thus, it follows that if (i) a KD produces high levels of ketones in mature intestinal epithelial cells (as Ang et al. [65] found) and (ii) these are not being generated in mature epithelial cells (as is suggested by the lack of HMGCS2 in the work of Chen et al. [88]), then the ketones are almost certainly coming from circulation. Along these lines, the authors write, “Because exogenous ketones rectify Hmgcs2 loss *in vitro* and *in vivo*, liver or other nonintestinal sources of ketones may substitute or supplement ISC-generated ketones in KTD-mediated regeneration, where circulating ketone levels are highly elevated” (88).

Lastly, it has recently been shown that not only can colonic epithelial cells oxidize both short- and long-chain fatty acids but also, through mitochondrial metabolism, these cells can oxidize medium- and long-chain acyl-carnitines that are delivered from biliary secretions (89). This has clinical relevance in that a reduction in these metabolites may contribute to colonic inflammation. Importantly, this provides yet another host-derived energy source for the epithelium during low-carbohydrate diets to complement the three discussed above. We will return to this below since bile acid secretion is a common concern with HF diets.

To conclude, we suggest that a more nuanced picture of how HF diets impact gut and overall health is required, with particular attention being paid to therapeutic KDs. By considering the alternative pathways by which ketones and KDs can influence gut function, we can move toward a more evolutionarily consistent picture of human gut variability. Nevertheless, research needs to clarify whether the benefits of lower carbohydrate or KDs come directly from increasing βHB, reducing inflammation, modifying insulin and glucose metabolism, reducing caloric intake, altering the gut microbiota, or other undetermined factors.

## HIGH-FAT DIETS, THE GUT, AND CRC— SETTLED SCIENCE?

We now briefly discuss colorectal cancer (CRC), where the HF diet-microbiota link is commonly highlighted. While the 2019 consensus statement by the International Cancer Microbiome Consortium acknowledges the need for better human studies into how the microbiota influences carcinogenesis (16), it nevertheless implicates a high-fat, low-fiber Western-style diet in changes in mucosal biomarkers of cancer risk (90). This follows the WHO’s 2015 classification of “red and processed meat” as a (class 2A) probable carcinogen, which relies heavily on preclinical and mechanistic data due to our current inability to isolate the effects of individual foods in clinical epidemiological studies (91, 92). A recent review in *Nature* echoes the WHO’s position on processed and red meat and cancer, aiming to establish an “oncogenic” CRC-associated microbiota (93).

In their *Nature* review, Janney et al. rely on the finding that CRC etiology is largely environmental, potentially accounting for 70 to 90% of the disease risk (93). Here, “diet” is strongly implicated, and the authors stress those diets that are low in fiber and high in fat and “red meat.” However, in the two references used to support this claim, only one mentions “red meat” (94) and only in the context of epidemiological associations, which are often plagued by healthy-user bias and significant reporting error (95, 96). Moreover, Janney et al. (93) vacillate between the terms “high-fat” and “Westernized high-fat,” which introduces confounders. Some mechanistic studies in mice do appear to support this HF diet-cancer link (97–100), but this might be tumor type specific (101, 102). As the “HF diets” studied are admixtures of refined sugars and/or hydrogenated oils, as mentioned above, this conflates a junk food-mimicking diet with any diet high in fat. Such results thus remain inconclusive (103), as we discuss below. Some of the suggested mechanisms connecting diet and CRC are protein fermentation, secondary bile acids, and increased levels of reactive oxygen species and reactive nitrogen species due to increased bile acid, heme iron, decreases in SCFAs, and specific microbial changes, e.g., an increased *Bilophila* abundance. As discussed above and explored in more detail below, the first three mechanisms are not clearly pathogenic, the putative antitumorigenic properties of butyrate (104) can also be obtained through alternative mechanisms, the relevance of heme iron remains to be seen (105, 106), and the significance of microbial changes depends on the broader physiological context and the relative abundances of microbes.

Since various conditions, such as irritable bowel syndrome (IBS), ulcerative colitis, and Crohn’s disease, appear to increase one’s risk for developing CRC (107–109), further research might consider the role of KDs in these contexts (110). Unfortunately, few such studies have been performed, and we are often left with case studies (111, 112).

We can, however, piece together various strands of evidence suggesting a more nuanced picture on animal fats as CRC risk factors. First, some mouse models and preclinical studies show KDs or ketones to be cancer suppressive (113, 114), perhaps primarily through glucose restriction (103) and by increasing intratumoral oxidative stress, leading to tumor cell apoptosis (115). Variations of therapeutic KDs might provide benefits for breast cancer by decreasing tumor necrosis factor alpha (TNF-α) and insulin while increasing interleukin 10 (IL-10) (116) and may be a promising adjuvant therapy for various cancers (117–121). However, as many of these studies are in animal models, caution is warranted (122, 123). At the least, these studies and recent reviews suggest a variety of mechanisms by which animal-food-based KDs may have beneficial effects on colorectal and other cancers.

This evidence converges with studies suggesting that reducing red meat and total fat consumption, while increasing fruit and grain consumption, does not reduce the risk for polyp reoccurrence even after 8 to 16 years (124–126) and has unclear risk benefits for CRC or any kind of cancer (127–129). Animal models suggesting that beef consumption does not promote cancer, that bacon may be protective, and that unsaturated fat may have carcinogenic effects (130, 131) all increase the likelihood that strong statements on animal foods and cancer are premature. Similarly, a growing number of reviews and meta-analyses weaken the links between meat consumption and cancer (132–136) and possibly overall health (137, 138), with some showing inverse correlations between meat intake and overall mortality in specific cohorts (139) and lower rates of CRC in meat eaters than in vegetarians (140). Studies that correlate meat intake with CRC also suggest a complex etiology due to contributing factors, such as obesity and hyperinsulinemia (141). As most of these studies have limitations, more research will be needed (142, 143), with the overall balance of evidence not currently appearing to support an independent effect of animal-based foods on the incidence of CRC. Given meat’s long-term presence in the hominid diet (144–147), it is more likely that modern dietary components and cooking techniques are driving cancer risk factors through their effects on our guts and general physiology.

## POSSIBLE OBJECTIONS AND CONCERNS

We acknowledge that there are likely to be various objections concerning the effects of fat and protein on our gut microbiota. We will address three of these, lipopolysaccharides, trimethylamine-*N*-oxide (TMAO), and secondary bile acids, and end with a cautionary note concerning KDs and hydrogen sulfide (H_2_S).

### LPS.

High-fat diets are commonly said to increase intestinal absorption of lipopolysaccharides (LPS), which are a group of endotoxins found in the cell walls of Gram-negative bacteria. If LPS gets into circulation, it can cause low-grade systemic inflammation (148), with the type and extent of the response dependent on the microbial source and LPS subtype (149). When we consume more long-chain fatty acids, our body makes more chylomicrons, which can carry LPS (150). Indeed, fat-enriched meals have been shown to moderately increase serum levels of LPS in both mice and humans (151, 152). While worth considering, we believe that, for several reasons, this is unlikely to contribute significantly to systemic inflammation in those consuming KDs.

First, several studies suggest that the transport of LPS by chylomicrons may confer an advantage because it favors the clearance of LPS by the liver, reducing LPS toxicity (153, 154). Moreover, chylomicrons have an innate ability to inactivate LPS (155), and the increased absorption of LPS appears to reduce inflammation in the gut mucosa (156). Similar beneficial adaptations can be seen with exercise, which increases LPS translocation but also LPS clearance, for instance via upregulation of anti-LPS immunoglobulins (157). This is important since the primary mode of systemic exposure to LPS is not through fat absorption but through reduced gut barrier function (158). When the gut is permeable, large amounts of LPS can leak into the submucosa and bloodstream, causing localized gut immune responses and systemic inflammation (159). This is likely to be a consequence, rather than a cause, of the metabolic endotoxemia associated with metabolic syndrome and cardiovascular disease, with poor systemic health subsequently impairing gut barrier function (160). Certain LPS subtypes have also been suggested to have beneficial immunomodulating roles (161). In other words, compared to the intestinal permeability associated with inflammatory gastrointestinal conditions, chylomicron-induced LPS absorption is likely minimal. One hypothesis emerging from these various studies that can be tested in humans is whether, for patients dealing with severe intestinal permeability, chylomicron-induced detoxification of LPS reduces inflammation enough to facilitate healing of the gut epithelium.

Importantly, many of the above-described studies are preclinical, but they nevertheless point to promising mechanisms that are being pursued in human studies to better contextualize the various roles of LPS. If fat-induced LPS absorption were an issue, we would expect to see increased systemic inflammation in those fed a KD. In contrast, humans consuming therapeutic KDs generally experience a reduction in systemic inflammation (162), with possible anti-inflammatory mechanisms, including NLRP3 inflammasome inactivation (163), modulation of TNF-α, IL-6, IL-8, MCP1, E-selectin, I-CAM, and PAI-1 (all studied in a registered clinical trial [164]), and an improved cytosolic NADH/NAD^+^ ratio (165). Taken together, these studies should assuage some concerns of LPS absorption following fat intake.

### TMAO.

Conventional nutrition science has long considered diets rich in animal-based foods a risk factor for cardiovascular disease. A recent mechanism of interest is TMAO (61). Increased concentrations of TMAO in circulation have been shown to contribute to atherosclerosis in animal models and correlate with cardiovascular disease risk in human studies (166). Certain gut bacteria convert choline and carnitine, both prominent in animal foods, to trimethylamine (TMA), which is then absorbed and oxidized in the liver to TMAO. However, some *in vitro* and animal evidence points to an altered small intestinal microbiota characterized by an overabundance of choline-consuming, TMA-producing Escherichia coli as the culprit for high TMAO, rather than excess consumption of animal products (167).

More importantly, a recent study suggests that gut microbiota composition can influence the amount of TMAO produced with an animal-based diet. Bacteria in the genus *Bilophila*, which tend to increase in subjects on an animal-based diet, may be able to help circumvent TMAO production by degrading TMA to dimethylamine (DMA) (168). Further analysis revealed that in a human cohort, *Bilophila* was significantly more abundant in the microbiotas of healthy individuals than in those with cardiovascular disease. As such, *Bilophila*’s pathogenicity may be context dependent, and it may even be beneficial for mitigating cardiovascular disease. Additionally, recent Mendelian randomization studies have suggested that increased TMAO in those at risk of cardiovascular disease may be a consequence of metabolic dysfunction, rather than an independent risk factor for disease risk (169). For multiple reasons, we thus believe that TMAO may not be a significant independent contribution to cardiovascular disease, with gut and overall health more likely to be the critical drivers of any associations.

### Bile acids.

It is commonly argued that an HF diet might be detrimental to the gut microbiota and gut barrier because it stimulates increased secretion of secondary bile acids (170). While some studies have shown that sustained exposure of the gut barrier to high concentrations of bile acids (above 400 μM) results in intestinal permeability (171), physiologic doses of bile acids (which may be nontoxic up to 50 to 100 μM [172]) have several potential benefits. For instance, bile acids have been shown to support barrier function by inducing the secretion of mucus from goblet cells, promoting epithelial cell migration, and boosting gut innate immune defenses (173). They can have antimicrobial properties, helping to regulate the gut microbiota, and may protect against small intestinal dysbiosis (174, 175). Several studies even suggest that bile acids activate enteroendocrine cells to release serotonin, which helps promote gut motility (176). Evidence for the physiological role of biliary secretions in producing an alternative fuel (acyl-carnitines) for gut epithelial cells was discussed above (89).

Exploring the complexities of every type of conjugated and deconjugated bile acid is beyond the scope of this article (177), but this should be sufficient to question the assumption that bile acid secretion resulting from the consumption of animal foods is inherently pathogenic.

### H_2_S.

There is one important caveat concerning KDs and individuals with H_2_S-associated bacterial overgrowth. H_2_S is normally produced in the body and acts as an important signaling molecule. Certain gut bacteria can also produce H_2_S, which at low concentrations has been shown to protect the gut against injury, stimulate gut motility, and support ulcer healing (178). However, an overabundance of these bacteria can lead to excess H_2_S, which has been linked to diarrhea, gut hypersensitivity, IBS, irritable bowel disease (IBD), and colorectal cancer (179), thereby suggesting pleotropic and dose-dependent effects (178). Some of the common H_2_S producers in the human gut, *Desulfovibrio* spp., Bilophila wadsworthia, and Fusobacterium nucleatum, tend to thrive on a diet that is high in animal protein and fat (180, 181). Thus, in patients with an overabundance of these microbes, it is probably best to avoid a ketogenic or high-fat diet until they can address this issue. Adding fiber to the diet (e.g., *Brassica* vegetables) may reduce the abundance of sulfate-reducing bacteria (182), further suggesting that the dietary context accompanying protein and fat consumption be considered.

Overall, we do not believe that there is sufficient evidence to suggest that the production of TMAO or LPS following animal protein/fat consumption or the physiologic increase of bile acids seen on a KD is harmful to the gut microbiota or gut barrier function. These metabolites might exacerbate ongoing pathological conditions of dysbiosis, but there are reasons to believe that they are not harmful under physiological conditions.

## LOOKING AHEAD: REDIRECTING RESEARCH FOR NUANCE

We conclude by suggesting how nutritional microbiota research might proceed in terms of what questions need to be asked or answered and how studies could be carried out.

1. Researchers should be more explicit about the kind of HF diets used. A diet mimicking a Western diet is not nutritionally equivalent to all HF diets, which vary in terms of fat sources and overall diet quality. Even changing the language in articles from “high-fat” to “high-fat, high-sugar” would better reflect the diet studied and could alter perceptions. From there, it will be helpful to study levels of fiber in HF or animal-based diets, which represent variations on the theme of nutritionally replete low-carbohydrate or therapeutic KDs.
2. While studies are starting to focus on different fat sources (4, 183, 184), still more are needed with as few confounders as possible. Some of the most problematic for mechanistic studies include the use of refined/hydrogenated fats and seed oils, which likely have rather different metabolic effects than fats from whole plant and animal foods, especially when these oils are mixed with refined sugars. Nutritional epidemiology should not only account for fat sources and dietary patterns/context but should explicitly address (un)healthy-user biases. The problem is not that such biases exist, as some may be unavoidable. The problem is that they remain under-discussed.
3. We should clearly acknowledge the limitations of animal research to inform human health/nutrition and our limited knowledge of what constitutes a “healthy” gut microbiota. Since it is likely that gut health encompasses more variability than is often acknowledged, we need to further test the evidence that humans evolved to tolerate, adapt to, and perhaps thrive on a variety of dietary patterns, with varying proportions of fiber, protein, and fat. An interdisciplinary approach may better elucidate the health effects of diet-gut interactions.
4. Researchers should explicitly state whether their interpretation considers the physiological context or makes claims based solely on isolated mechanisms and nutritional epidemiology. Properly labeling evidence and placing observations in the broader context of research can help prevent potentially biased interpretations.
5. Similarly, researchers might carefully consider the differences in host and microbial metabolism. For instance, studies relying on fecal samples might skew towards microbes found in the large intestine or colon, where carbohydrates are digested and metabolized. This may obscure the role of microbial and host responses in the small intestine (9, 185), thereby underrepresenting the microbes that are more involved in fatty acid catabolism or the production of lipase coenzymes in the jejunum. Similarly, by focusing largely on microbial metabolism of carbohydrates and fiber, we might be overlooking host-specific metabolism, which appears to be highly adaptable to relative levels of dietary fat and protein.
6. Clinicians can also remain open-minded to alternative dietary approaches. One implication of considering alternative energy sources is that in the presence of a “healthy” microbiota and gut mucosa, butyrate is probably sufficient to fuel the gut. However, if patients (i) have ulcerative colitis or other mucosal damage, with impaired butyrate uptake, (ii) have gut dysbiosis characterized by a lack of butyrate producers, or (iii) are on a restrictive diet, such as a low-FODMAP (fermentable oligosaccharides, disaccharides, monosaccharides, and polyols) diet or the specific carbohydrate diet (SCD), resulting in reduced butyrate production, it may be wise for clinicians to consider “nontraditional” therapeutic options, such as KDs, to support gut epithelial metabolism, at least until treating the underlying gut pathologies and healing the gut mucosa.
7. Finally, it will be important to objectively weigh the evidence concerning plants, animal fats, and proteins. Conventional wisdom holds the belief that plants are definitively healthful and animal products are at least potentially harmful. The effects of this belief can lead to conflicts of interest in nutrition studies more generally (186) and may influence the decision-making leading to dietary guideline statements that provide strong recommendations despite abundant evidence supporting the idea that humans can thrive on a diverse range of diets (187).

In the end, we hope that more time and research will help to uncover these biases and lead to a more accurate depiction of the responsiveness of the human gut and its microbes to nutritional variations.
